# The Vicious Cycle of Diabetic Kidney Disease, Vitamin D Deficiency, and Arterial Hypertension

**DOI:** 10.3390/healthcare14050662

**Published:** 2026-03-05

**Authors:** Barbara Kurzyna, Patrycja Czebreszuk, Wiktoria Szczerbińska, Bartłomiej Michalak, Maciej Walędziak, Anna Różańska-Walędziak

**Affiliations:** 1 Faculty of Medicine, Collegium Medicum, Cardinal Stefan Wyszynski University in Warsaw, 01-938 Warsaw, Poland; b.kurzyna1@gmail.com (B.K.); ppczebreszuk@gmail.com (P.C.); szczerbinska.wiktoria@gmail.com (W.S.); 2 Department of General, Oncological, Metabolic and Thoracic Surgery, Military Institute of Medicine–National Research Institute, 04-141 Warsaw, Poland; maciej.waledziak@gmail.com; 3 Department of Human Physiology and Pathophysiology, Faculty of Medicine, Collegium Medicum, Cardinal Stefan Wyszynski University in Warsaw, 01-938 Warsaw, Poland; aniaroza@tlen.pl

**Keywords:** arterial hypertension, diabetic kidney disease, insulin resistance, proteinuria, renin–angiotensin–aldosterone system, vitamin D deficiency, vitamin D metabolism, vitamin D supplementation

## Abstract

Diabetic kidney disease (DKD) is a major complication of diabetes mellitus that contributes substantially to chronic kidney failure and increased cardiovascular risk. Beyond progressive deterioration of renal function, DKD is associated with disturbances in endocrine and vascular regulation. Among these, alterations in vitamin D homeostasis and blood pressure (BP) control represent clinically relevant, yet incompletely integrated aspects of DKD pathophysiology. This narrative review synthesizes current evidence on the multidirectional relationships between DKD, vitamin D deficiency, and arterial hypertension (AH). Attention is given to renal mechanisms responsible for reduced vitamin D availability in DKD, including proteinuria-related loss of vitamin D-binding proteins, impaired proximal tubular reabsorption, decreased renal activation of vitamin D, and hormonal regulators such as fibroblast growth factor-23. It further discusses how insufficient vitamin D signaling may influence renal and vascular pathways involved in BP regulation. Mechanistic links between vitamin D deficiency and AH in DKD are discussed, with emphasis on maladaptive activation of the renin–angiotensin–aldosterone system (RAAS), persistent inflammation, oxidative stress, endothelial dysfunction, and insulin resistance. These interdependent processes promote both renal injury progression and sustained elevations in BP, forming a self-reinforcing pathogenic loop. Finally, available data on vitamin D-based therapeutic strategies in DKD are reviewed, including native vitamin D supplementation, active vitamin D metabolites, and vitamin D receptor agonists. Although experimental and observational studies suggest potential nephroprotective and vasculoprotective effects, evidence from randomized clinical trials remains heterogeneous. Further well-designed prospective studies are required to clarify the clinical utility of vitamin D interventions in patients with DKD and coexisting AH.

## 1. Introduction

Diabetic kidney disease (DKD) represents a complex and progressive microvascular complication of diabetes mellitus that arises from the long-standing interplay between metabolic dysregulation, hemodynamic stress, and inflammatory signaling. Chronic hyperglycemia induces structural and functional alterations in the kidney, resulting in persistent albuminuria and/or a gradual decline in glomerular filtration rate (GFR). DKD affects approximately 30–40% of patients with diabetes and remains a leading cause of end-stage renal disease requiring renal replacement therapy. As a renal manifestation of diabetes-related microangiopathy, DKD constitutes the predominant clinical subtype of chronic kidney disease (CKD) and shares core pathophysiological mechanisms with CKD. As DKD advances, abnormalities of the glomerular and tubular compartments extend beyond localized tissue injury and contribute to systemic disturbances that promote arterial hypertension (AH) and disrupt cardiovascular homeostasis [1,2].

One of the potentially key, yet often underrecognized consequences of progressive renal damage in DKD is impairment of vitamin D metabolism and signaling, as the kidney plays a central role in maintaining vitamin D homeostasis [3]. Damage to glomeruli and proximal tubules, therefore, directly compromises vitamin D availability and biological activity, predisposing patients with DKD to both quantitative and functional vitamin D deficiency [1,2].

Definitions of vitamin D deficiency vary across expert groups. The Food and Nutrition Board (FNB) of the National Academies of Sciences, Engineering, and Medicine considers serum 25-hydroxyvitamin D [25(OH)D] concentrations below 30 nmol/L (12 ng/mL) to indicate deficiency, 30–50 nmol/L (12–20 ng/mL) to reflect potential inadequacy, and concentrations ≥ 50 nmol/L (≥20 ng/mL) to be sufficient for most individuals [4]. In contrast, the Endocrine Society Task Force on Vitamin D defines vitamin D deficiency as serum 25(OH)D levels below 50 nmol/L (20 ng/mL) [5]. Given the lack of universal consensus regarding optimal 25(OH)D concentrations, for the purposes of this review vitamin D deficiency is defined as serum 25(OH)D < 20 ng/mL (50 nmol/L).

Concurrently, DKD is closely linked to dysregulation of blood pressure (BP) control through mechanisms intrinsic to renal injury, most notably maladaptive activation of the renin–angiotensin–aldosterone system (RAAS) [6,7]. In parallel, vitamin D deficiency that develops in the course of DKD further contributes to BP dysregulation by impairing the physiological inhibitory effects on renin expression, inflammatory signaling, and endothelial function [5,8,9,10,11,12,13,14]. As a result, AH in DKD reflects the combined effects of kidney-derived mechanisms and impaired vitamin D signaling. It acts both as a consequence of renal dysfunction and as a driver of progressive kidney injury.

At the population level, vitamin D deficiency has been in the spotlight as a major public healthcare issue with an estimated prevalence of more than a billion people worldwide [12,15]. AH also constitutes a major global public health problem, affecting over 1.5 billion individuals worldwide and representing one of the leading modifiable risk factors for cardiovascular morbidity and mortality [13,16]. Its prevalence is particularly high among patients with diabetes and DKD [17].

Against this background, DKD, vitamin D deficiency, and AH should not be viewed as isolated clinical entities but rather as interconnected components of a vicious cycle in which renal injury disrupts vitamin D homeostasis, impaired vitamin D signaling contributes to BP dysregulation, and sustained hypertension further aggravates renal and cardiovascular damage.

The aim of this review is to examine the mechanisms linking DKD, vitamin D deficiency, and AH, and to explore their implications for disease progression and clinical management across these interrelated conditions.

## 2. Material and Methods

This narrative review was conducted to synthesize current evidence on the relationships between diabetic kidney disease, vitamin D deficiency, and arterial hypertension, with particular focus on pathophysiological mechanisms and clinical implications.

A structured literature search was performed in the PubMed/MEDLINE and Scopus databases. The search covered publications from 2017 to 2025 and was limited to peer-reviewed articles published in English. Search terms were combined using Boolean operators (AND, OR) and included: “diabetic kidney disease”, “vitamin D deficiency”, “vitamin D metabolism”, “vitamin D receptor”, “vitamin D supplementation”, “arterial hypertension”, “renin–angiotensin–aldosterone system”, “RAAS”, “endothelial dysfunction”, “oxidative stress”, “inflammation”, “insulin resistance”, and “proteinuria”. Additional sources were identified through manual screening of reference lists from selected key publications. All sources were last searched/consulted on 10 December 2025.

Eligible studies included randomized controlled trials, observational studies, clinical guidelines, systematic reviews, and relevant experimental or translational studies examining mechanistic or clinical links between DKD, vitamin D status, and hypertension. Preference was given to original research articles as well as recent high-quality systematic and narrative reviews providing comprehensive mechanistic or clinical insights. Mechanistic and experimental studies were included when they contributed to understanding the biological pathways involved. Publications were selected based on their novelty, methodological quality, and clinical relevance, with particular emphasis on those providing up-to-date data, clinical insight, or addressing areas of uncertainty.

Papers were excluded if they were case reports, conference abstracts without full text, non-peer-reviewed publications, or were not sufficiently relevant to the pathophysiological or clinical relationships among DKD, vitamin D status, and BP regulation. Both human and mechanistic animal studies were considered when relevant to the biological pathways discussed.

All retrieved records were screened independently by three researchers (B.K.; P.C.; W.S.) under the supervision of two senior investigators (A.R.-W.; M.W.). Duplicates were removed using database tools and manual verification. Screening was performed in two stages: title and abstract review followed by full-text assessment. Discrepancies were resolved through discussion and consensus; when necessary, a senior reviewer adjudicated disagreements.

The review was structured into thematic chapters covering: (1) mechanisms leading to vitamin D deficiency in DKD; (2) the nephroprotective role of vitamin D with emphasis on regulation of the RAAS; (3) mechanisms linking vitamin D deficiency to the development of AH in DKD, including RAAS overactivation, inflammation, oxidative stress, endothelial dysfunction, and insulin resistance; and (4) therapeutic implications of vitamin D supplementation in patients with DKD and AH.

Given the narrative design of this review, no formal risk-of-bias scoring or meta-analysis was conducted. However, study quality and methodological rigor were considered descriptively during evidence synthesis. Due to heterogeneity in study designs and reported outcomes, findings were summarized narratively to provide a structured qualitative overview of the available evidence.

## 3. Results

### 3.1. Why Does Vitamin D Deficiency Occur in Diabetic Kidney Disease?

**Figure 1:**
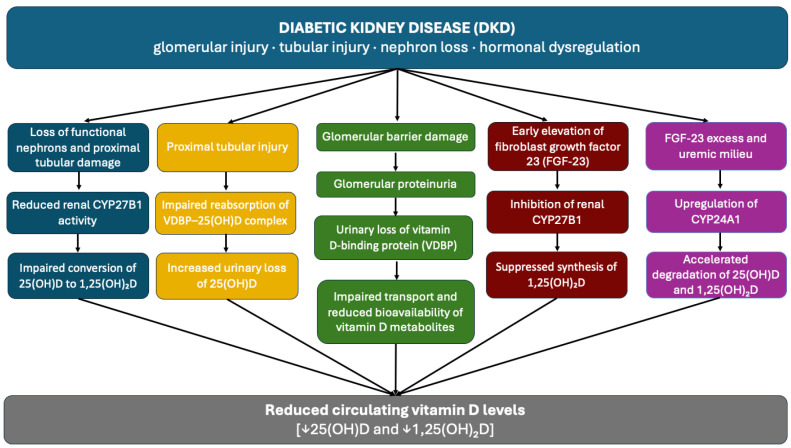
**Mechanisms linking diabetic kidney disease to reduced circulating vitamin D levels.** DKD disrupts vitamin D homeostasis through multiple, parallel mechanisms involving glomerular injury, proximal tubular damage, nephron loss, and hormonal dysregulation. Loss of functional nephrons and suppression of renal CYP27B1 activity impair the conversion of 25(OH)D to its active form, 1,25(OH)_2_D **(blue boxes)**. Proximal tubular injury reduces reabsorption of the VDBP–25(OH)D complex, limiting substrate availability **(yellow boxes)**, while glomerular proteinuria leads to urinary loss of VDBP and impaired transport of vitamin D metabolites **(green boxes)**. Early elevations in FGF-23 further inhibit vitamin D activation **(red boxes)** and promote CYP24A1-mediated catabolism **(pink boxes)**. Collectively, these processes converge to reduce circulating concentrations of both 25-hydroxyvitamin D and 1,25-dihydroxyvitamin D.

Observational and experimental studies have demonstrated that patients with DKD exhibit reduced circulating concentrations of both 25-hydroxyvitamin D [25(OH)D] and its biologically active form, 1,25-dihydroxyvitamin D [1,25(OH)_2_D], with these deficiencies becoming more pronounced as renal function declines [1,2,5,11]. These observations suggest that disturbances in vitamin D homeostasis represent a clinically relevant feature of DKD [1]. The following section, therefore, focuses on the renal and hormonal mechanisms linking glomerular and tubular injury to the development of vitamin D deficiency in DKD [Figure 1].

One of the earliest and most direct mechanisms contributing to vitamin D deficiency in DKD is the urinary loss of vitamin D-binding protein (VDBP) secondary to glomerular proteinuria [9]. In circulation, vitamin D is predominantly transported bound to VDBP, a low-molecular-weight protein of approximately 58 kDa [1,2,12,15]. In DKD, disruption of the glomerular filtration barrier results in proteinuria and increased urinary loss of VDBP [18]. Because VDBP serves as the primary carrier of 25(OH)D in plasma, its urinary loss may contribute to depletion of circulating vitamin D stores. Elevated urinary VDBP concentrations have been consistently reported in patients with kidney damage, highlighting proteinuria as a disease-specific contributor to vitamin D deficiency in DKD [2,11,18].

In addition to glomerular protein loss, tubular dysfunction is considered an important mechanism through which DKD disrupts vitamin D homeostasis [1]. Under normal conditions, the filtered VDBP–25(OH)D complex is efficiently reabsorbed in the proximal tubule. However, tubular injury characteristic of DKD compromises this reabsorptive process, leading to reduced recovery of filtered 25(OH)D and progressive depletion of vitamin D stores [1,2,12]. Consequently, tubular damage in DKD impairs not only solute handling but also the renal conservation of vitamin D metabolites, thereby contributing to systemic vitamin D deficiency [12].

DKD also compromises the renal enzymatic activation of vitamin D, as the kidneys represent the principal site of vitamin D activation. In the proximal renal tubules, the enzyme 1α-hydroxylase (CYP27B1) catalyzes the conversion of 25(OH)D into its biologically active form, 1,25(OH)_2_D, underscoring the dependence of vitamin D activation on preserved renal tubular structure and function [13]. In CKD, including DKD, progressive loss of functional nephrons and tubular injury impair the kidney’s capacity to activate vitamin D. Damage to proximal tubular cells leads to reduced CYP27B1 activity, thereby limiting the conversion of circulating 25(OH)D into its active form and resulting in a decline in circulating 1,25(OH)_2_D concentrations [1,2,11,13,18]. Notably, a marked reduction in 1,25(OH)_2_D levels has been observed particularly when GFR declines to 40 mL/min or below [2].

In parallel with structural alterations, hormonal dysregulation further contributes to impaired vitamin D metabolism in DKD. Vitamin D metabolism is tightly regulated by mineral homeostasis-related hormones, including fibroblast growth factor 23 (FGF-23). FGF-23 acts as a potent inhibitor of renal CYP27B1 while simultaneously inducing 24-hydroxylase (CYP24A1), thereby suppressing the synthesis of 1,25(OH)_2_D and promoting its catabolism [2,9,13,15,19]. In DKD, elevated FGF-23 levels observed early in the disease course further reduce the availability of active vitamin D, even in the presence of residual renal function [8]. Increased CYP24A1 expression has also been reported in diabetic patients and experimental models of uremia, which additionally contribute to reduced concentrations of both 25(OH)D and 1,25(OH)_2_D, supporting the concept of a progressive nature of vitamin D deficiency in DKD [1,2,11].

Given the high prevalence of vitamin D deficiency among patients with DKD and its association with impaired renal structure, function, and endocrine activity, the mechanisms outlined above provide a mechanistic basis for reduced vitamin D-mediated nephroprotection [2,15,18]. This impairment may contribute to progressive renal injury and, consequently, to the development of AH [8,11,12], as discussed in the following section.

### 3.2. How Does Vitamin D Deficiency Lead to Loss of Nephroprotection Through RAAS Overactivation in DKD?

In DKD, chronic activation of the RAAS is widely regarded as a central mechanism implicated in progressive renal injury rather than a purely physiological regulator of BP [20,21]. Beyond its systemic hemodynamic effects, the RAAS is integral to the regulation of renal activity [21]. Sustained RAAS activation is associated with a cascade of pathogenic events, including renal inflammation, podocyte injury, endothelial dysfunction, disruption of the glomerular filtration barrier, glomerulosclerosis, and progressive renal fibrosis [20].

Renin, encoded by the REN gene and predominantly synthesized and secreted by juxtaglomerular cells of the kidney, catalyzes the rate-limiting step of RAAS activation by converting angiotensinogen into angiotensin I (Ang I) [22]. Angiotensin II (Ang II), generated downstream via angiotensin-converting enzyme, exerts potent vasoconstrictive and profibrotic effects through activation of the angiotensin II type 1 receptor (AT1R) [23]. Tightly controlling REN gene expression is therefore critical to maintaining renal structure and function, which, in turn, helps maintain normotension [21,22].

Vitamin D deficiency may be associated with a loss of nephroprotective restraint on RAAS by disrupting suppression of cAMP response element-binding protein (CREB) activity mediated by the vitamin D receptor (VDR), contributing to increased renin expression and excessive Ang II production [22,24]. This mechanism has been demonstrated primarily in experimental models, whereas human data remain largely indirect. Transcriptional regulation of renin is largely mediated by the CREB, which promotes REN gene expression through its interaction with cAMP response elements (CREs) located within the proximal promoter and enhancer regions of the renin gene. CREB activity is modulated by 1,25(OH)_2_D, the biologically active form of vitamin D, via activation of the nuclear VDR. Experimental evidence from murine models indicates that ligand-activated VDR directly interacts with CREB, thereby preventing its binding to CRE sites and suppressing renin transcription [22]. However, translation of these findings into consistent clinical effects in DKD patients has not been uniformly confirmed.

Beyond CREB-mediated regulation of renin transcription, the kidney represents a major target for vitamin D due to its high expression of VDRs. These nuclear receptors modulate key pathogenic pathways implicated in DKD progression, including inflammation, oxidative stress, and fibrosis. Experimental studies in diabetic animal models have demonstrated that VDR deficiency leads to upregulated expression of renin, angiotensin, and angiotensin receptors, resulting in excessive intrarenal RAAS activation. This dysregulation precipitates severe renal injury [20]. Albuminuria is a well-established early clinical marker of DKD and reflects underlying glomerular damage driven in part by RAAS overactivation [25,26].

The clinical efficacy of RAAS blockade in reducing albuminuria and slowing disease progression further supports the important role of RAAS dysregulation in DKD [25,26]. Calcitriol and vitamin D analogues such as paricalcitol suppress renin and angiotensinogen expression in renal cells [20,27]. These observations indirectly support the concept that loss of effective vitamin D signaling in DKD removes an important physiological restraint on RAAS activity.

Thus, in DKD, vitamin D deficiency manifests not as an isolated metabolic abnormality but as a functional loss of nephroprotection, leading to RAAS overactivation. This RAAS-centered mechanism provides the upstream trigger for many of the hemodynamic and non-hemodynamic processes discussed in subsequent sections, creating a self-perpetuating pathogenic loop in which excessive RAAS activity accelerates renal injury and promotes progressive kidney damage, including the development of AH [20,21,24].

### 3.3. Mechanisms Linking Vitamin D Deficiency to the Development of Arterial Hypertension in DKD Patients

#### 3.3.1. RAAS Overactivation and Local Ang II Accumulation

The inhibitory effect of vitamin D on RAAS activity is supported by experimental and some clinical data, although the strength of evidence varies across study designs [5]. In DKD, vitamin D deficiency may further exacerbate the diabetic milieu, characterized by persistent hyperglycemia, oxidative stress, and chronic inflammation, which synergistically amplify both systemic and intrarenal RAAS activity [20]. Excessive Ang II production is associated with a cascade of pathogenic events characterized by potent vasoconstriction, enhanced tubular sodium retention, increased aldosterone secretion, and subsequent extracellular fluid volume expansion [28,29].

While the systemic consequences of RAAS overactivation have been outlined above, local dysregulation of the intrarenal RAAS represents an additional pathogenic mechanism in DKD. Hyperglycemia directly stimulates renin and Ang II synthesis within mesangial cells, leading to local Ang II accumulation independent of systemic RAAS activity [30]. Intrarenal Ang II profoundly alters glomerular hemodynamics, as the efferent arteriole is markedly more sensitive to Ang II-induced vasoconstriction than the afferent arteriole. This imbalance elevates intraglomerular capillary pressure [31].

Regardless of its systemic or local origin, excess Ang II promotes both hemodynamic and non-hemodynamic effects that drive renal and cardiovascular pathology.

The hemodynamic consequences of RAAS overactivation play a central role in the development of AH. Ang II-mediated vasoconstriction and enhanced tubular sodium reabsorption may increase extracellular fluid volume and contribute to sustained BP elevation [29]. Clinically, this pathological RAAS-driven BP elevation meets the diagnostic criteria for hypertension, commonly defined as systolic blood pressure ≥ 140 mmHg and/or diastolic blood pressure ≥ 90 mmHg in office-based measurements according to the International Society of Hypertension (ISH) Global Hypertension Practice Guidelines. It should be noted that diagnostic thresholds and treatment targets may differ in patients with chronic kidney disease or diabetes, in whom pharmacological therapy is typically initiated at ≥140/90 mmHg, while recommended blood pressure targets are <130/80 mmHg (or <140/80 mmHg in elderly individuals) [32]. Chronic hypertension imposes excessive mechanical stress on the vascular endothelium, contributing to progressive vascular injury [33]. Within the kidney, prolonged hypertension induces ischemic injury of the renal vasculature, glomeruli, and tubulointerstitial, culminating in nephrosclerosis and interstitial fibrosis [34,35]. Renal ischemia further stimulates Ang II synthesis, thereby perpetuating RAAS overactivation and renal damage [34].

Beyond its hemodynamic actions, sustained Ang II signaling exacerbates renal injury through non-hemodynamic mechanisms, including stimulation of renal cell hypertrophy, induction of pro-inflammatory cytokines, enhanced extracellular matrix deposition, and macrophage infiltration, collectively accelerating glomerulosclerosis and tubulointerstitial fibrosis [30,36]. Non-hemodynamic effects also include Ang II-induced aldosterone secretion, which activates the mineralocorticoid receptor (MR), abundantly expressed in renal and vascular tissues. Chronic MR overactivation promotes inflammation and fibrosis, further reinforcing renal structural damage and cardiovascular remodeling [28]. These mechanisms are well established experimentally. However, their modulation by vitamin D in humans remains incompletely characterized.

**Figure 2:**
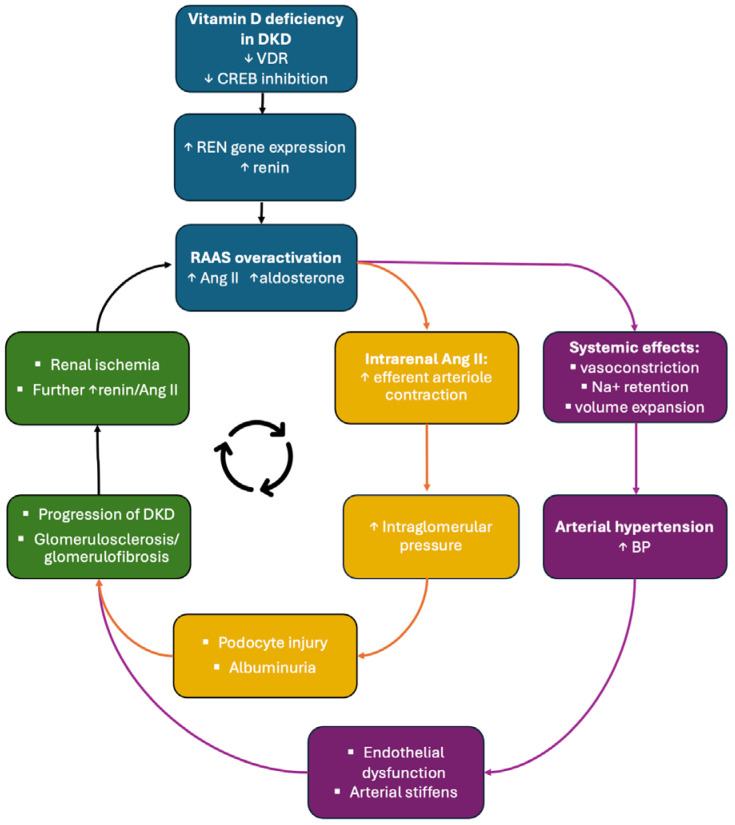
**The vicious cycle linking vitamin D deficiency, RAAS overactivation, DKD, and AH.** Vitamin D deficiency leads to reduced activation of the VDR, resulting in loss of CREB-mediated suppression of renin transcription and subsequent RAAS overactivation. Excessive Ang II promotes intrarenal hemodynamic disturbances, fibrosis, and albuminuria, accelerating DKD progression. RAAS-driven arterial hypertension further exacerbates renal ischemia and injury, reinforcing RAAS activation and perpetuating the pathogenic cycle. Upward arrows (↑) indicate an increase or upregulation, whereas downward arrows (↓) indicate a decrease or suppression of the respective process, mediator, or physiological parameter.

As renal function declines, RAAS activity becomes increasingly upregulated, creating a cycle [Figure 2] in which hypertension both drives and results from progressive kidney injury [29].

#### 3.3.2. Chronic Inflammation and Oxidative Stress

Chronic inflammation and oxidative stress are widely considered interrelated contributors to the pathogenesis of DKD and its cardiovascular complications. Chronic hyperglycemia is regarded as an important driver of these processes, as it induces profound metabolic disturbances in endothelial cells, vascular smooth muscle cells, renal tubular cells, and mesangial cells. Persistently elevated glucose levels are thought to promote both microvascular and macrovascular injury through several interrelated pathways [37].

These pathways include enhanced glycoxidation, increased intracellular generation of reactive oxygen species (ROS), and accumulation of glycated proteins. Excessive ROS production is associated with disruption of cellular redox balance and may amplify inflammatory signaling, while progressive impairment of endogenous antioxidant systems may sustain this pathological state [38].

As a result, pro-inflammatory signaling becomes chronically activated, leading to persistently elevated circulating levels of inflammatory mediators, including IL-1, IL-6, CRP, MCP-1, and TNF-α. This pattern is consistent with the view of DKD as a metabolically driven immune-inflammatory disorder. Consistently, elevated levels of systemic inflammatory markers, such as high-sensitivity C-reactive protein (hs-CRP), have been associated with disease progression in patients with type 2 diabetes mellitus (T2DM), though causality remains difficult to establish [39]. The consequences of sustained inflammation and oxidative stress likely extend beyond the kidney. Mitochondrial dysfunction develops not only in renal and vascular cells but also in pancreatic β-cells, where it may contribute to impaired insulin secretion and worsening glycemic control [37].

An important mechanistic link between oxidative stress and vitamin D signaling in this context is glutathione (GSH), the major intracellular antioxidant. Experimental data suggest that vitamin D may contribute to redox homeostasis in part by upregulating intracellular GSH levels and antioxidant enzyme activity, thereby limiting excessive ROS generation. At the same time, adequate GSH availability appears necessary for optimal bioavailability and intracellular signaling of vitamin D metabolites. In diabetes, and particularly in DKD, chronic oxidative stress is associated with GSH depletion, which may compromise vitamin D signaling, exacerbate IR, and further weaken endogenous antioxidant defenses. Although this reciprocal interaction has been described primarily in selected clinical studies, it provides a plausible framework for understanding how redox imbalance and altered vitamin D status may interact [40,41].

As these redox and inflammatory disturbances extend beyond the cellular level, they may manifest as systemic vascular alterations with clinically relevant hemodynamic consequences. Both experimental and clinical studies suggest an association between inflammatory and redox-related pathways in the development of AH. Prospective cohort and meta-analytic data indicate that elevated inflammatory markers are associated with a 20-40% increased risk of incident hypertension, while biomarkers of oxidative stress and reduced antioxidant capacity consistently correlate with higher BP levels [42,43]. These observations suggest that inflammatory and redox-related mechanisms may converge at the level of the vascular endothelium, rather than acting as independent parallel pathways.

#### 3.3.3. Endothelial Dysfunction

In DKD, endothelial dysfunction represents a common pathway through which chronic metabolic, inflammatory, and hormonal disturbances are translated into vascular injury and dysregulation of BP [44,45]. In this setting, endothelial dysfunction provides the functional basis for progressive structural vascular alterations, leading to increased peripheral resistance and hypertension.

Vascular homeostasis is maintained by endothelial cells through the tightly coordinated release of vasoactive mediators that balance vasodilation and vasoconstriction. Nitric oxide (NO) plays a central role in this process by promoting vasodilation, limiting vascular smooth muscle cell contraction, and reducing peripheral vascular resistance, thereby supporting normal BP regulation [46]. In contrast, endothelin-1 (ET-1) exerts potent vasoconstrictive effects and functionally opposes NO-mediated vasodilation. The balance between NO and ET-1 is regarded as critical for maintaining vascular tone and resistance [38]. Loss of this vasomotor equilibrium compromises endothelial regulation of vascular tone. Reduced NO availability not only limits vasodilatory capacity but also favors a shift toward vasoconstriction. In this context, the combined actions of NO, ET-1, and oxidative stress have been implicated in processes extending beyond altered vascular reactivity to influence extracellular matrix synthesis by vascular smooth muscle cells and fibroblasts. This interaction is thought to promote fibrotic remodeling of the vascular wall and progressive arterial stiffening potentially contributing to sustained increases in vascular resistance and the development of AH [38,43,47].

Chronic hyperglycemia is considered an additional driver of endothelial injury in DKD. Sust1ained elevations in glucose favor the formation and accumulation of advanced glycation end products (AGEs) within the vascular wall. AGEs have been shown to impair endothelial integrity by reducing NO bioavailability, enhancing inflammatory signaling, and increasing endothelial permeability, thereby compromising renovascular function and promoting vasoconstrictive responses [34,37,39].

As endothelial dysfunction persists, these functional abnormalities translate into structural remodeling of the arterial wall. Continuous endothelial injury has been associated with thickening of the vessel wall and a gradual loss of arterial elasticity. As large and medium-sized arteries stiffen, their ability to dilate with each cardiac cycle diminishes, leading to elevated systolic BP and increased cardiac workload. Arterial stiffness is therefore considered a key mechanistic link between sustained endothelial dysfunction and the development and progression of AH in patients with DKD.

Against this background, vitamin D deficiency has been proposed as an important amplifying factor that exacerbates endothelial dysfunction and contributes to the transition from functional vascular impairment to overt hypertension. Vitamin D supports endothelial function primarily by maintaining NO bioavailability [46]. In the setting of vitamin D deficiency, NO availability may be reduced, due to impaired nitric oxide synthase activity and enhanced oxidative inactivation of NO, leading to attenuated NO-dependent vasodilation [46,48]. At the same time, vitamin D deficiency has been associated with increased ET-1 activity, further shifting the vasomotor balance toward vasoconstriction. The combined effect of reduced NO-mediated vasodilation and enhanced ET-1-dependent vasoconstriction may lead to sustained increases in peripheral vascular resistance and may favor the development of AH, particularly in patients with CKD and DKD [37].

#### 3.3.4. Insulin Resistance

Observational studies have shown inverse associations between serum 25(OH)D levels and markers of IR, such as HOMA-IR, suggesting that vitamin D deficiency may impair insulin sensitivity. This metabolic abnormality has been associated with the development of AH, particularly in patients with DKD [49].

At the molecular level, vitamin D has been reported to modulate insulin signaling through both genomic and non-genomic mechanisms, including regulation of insulin receptor substrate (IRS) transcription and calcium flux. Activation of the vitamin D receptor has been shown in experimental models to influence the transcription of genes involved in insulin action, including the insulin receptor and IRS. Experimental data indicate that vitamin D supplementation may increase IRS expression, enhancing insulin sensitivity in peripheral tissues [40]. The presence of vitamin D response elements within the promoter region of the insulin receptor gene further supports a direct regulatory role of vitamin D in insulin signaling [41].

IR is thought to contribute to vascular dysfunction through impaired endothelial signaling. Insulin stimulates endothelial NO production while suppressing ET-1 secretion. In IR states, this balance may shift toward systemic and renal vasoconstriction. Reduced NO bioavailability also promotes renal vasoconstriction and increases sodium reabsorption, thereby contributing to elevated BP [50]. Vitamin D is also involved in the regulation of intracellular calcium homeostasis, which is important for insulin signal transduction and glucose uptake in insulin-sensitive tissues. Vitamin D deficiency can impair glucose transporter activity and thereby exacerbate peripheral IR [49]. Moreover, vitamin D has been reported to influence calcium-dependent insulin secretion in pancreatic β-cells via both direct binding to the vitamin D receptor and indirect modulation of calcium flux, influencing systemic insulin sensitivity [51,52].

In DKD patients, IR has been linked to elevated BP through hemodynamic mechanisms, including hyperinsulinemia-induced activation of the sympathetic nervous system, increased cardiac output, enhanced peripheral vascular resistance, and stimulation of vascular smooth muscle proliferation and endothelin synthesis. Additionally, IR has been associated with activation of RAAS, promoting sodium retention and further elevation of blood pressure [53,54].

**Figure 3:**
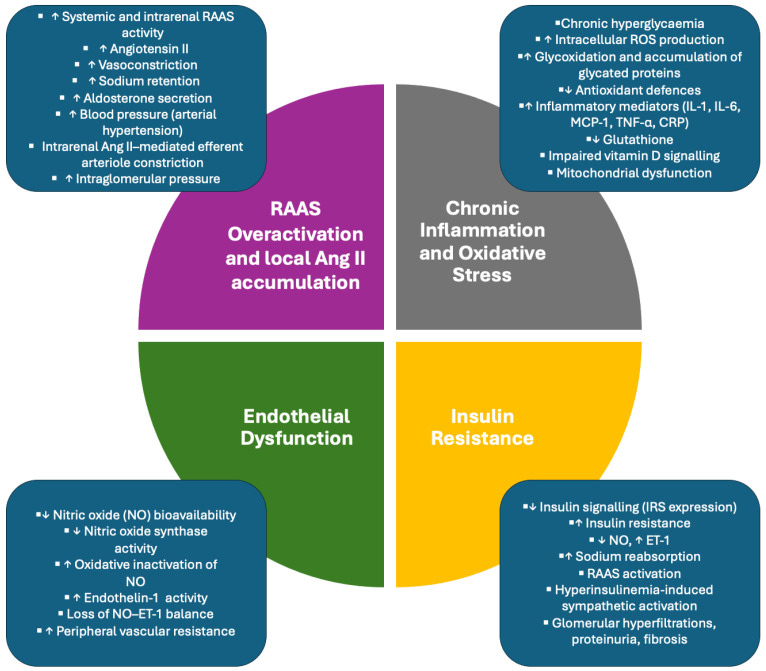
**Interplay between vitamin D deficiency, DKD, and key mechanisms driving AH.** Vitamin D deficiency contributes to reduced inhibition of RAAS, leading to increased systemic and intrarenal RAAS activity and elevated Ang II levels. Excess Ang II promotes vasoconstriction, sodium retention, aldosterone secretion, and intraglomerular hypertension through preferential efferent arteriole constriction. In parallel, chronic hyperglycemia induces oxidative stress and chronic inflammation, characterized by increased reactive oxygen species production, glycoxidation, depletion of antioxidant defenses, and mitochondrial dysfunction. These processes converge on endothelial dysfunction, marked by reduced nitric oxide bioavailability, increased ET-1 activity, loss of vasomotor balance, and elevated peripheral vascular resistance. Vitamin D deficiency further exacerbates insulin resistance, impairing insulin signaling and endothelial nitric oxide production, activating RAAS and sympathetic pathways, and promoting renal hyperfiltration and fibrosis. Collectively, these interrelated mechanisms create self-perpetuating loops that accelerate vascular dysfunction, progressive kidney injury, and the development of AH in patients with DKD. Upward arrows (↑) indicate an increase or upregulation, whereas downward arrows (↓) indicate a decrease or suppression of the respective process, mediator, or physiological parameter.

Beyond its hemodynamic effects, IR has been implicated in the progression of kidney damage. Hyperinsulinemia has been associated with glomerular hyperfiltration, increases vascular permeability, and promotes proteinuria, contributing to nephron loss and renal fibrosis [53,55]. Neurohormonal activation and increased efferent arteriolar tone elevate intraglomerular pressure, leading to podocyte injury and accelerating DKD progression [56]. Furthermore, RAAS dysregulation has been proposed to aggravate insulin resistance by increasing angiotensin II-mediated oxidative stress and promoting proteasome-mediated degradation of IRS-1, impairing insulin signaling [57]. Taken together, vitamin D deficiency may represent one of several mechanistic links connecting DKD, IR, and AH [Figure 3].

### 3.4. Therapeutic Implications of Vitamin D Supplementation

Over the past decade, assessment of vitamin D status and prescription of vitamin D supplementation have become routine in clinical practice. This growing interest reflects the expectation that correction of vitamin D deficiency may confer benefits beyond mineral metabolism. However, in the absence of robust evidence, widespread testing and supplementation may contribute to clinical uncertainty. In patients with CKD, particularly those with DKD, vitamin D deficiency frequently coexists with AH, raising a clinically relevant question: does such supplementation represent a meaningful therapeutic strategy in this setting, or does it remain an attractive yet unproven intervention [5]?

Vitamin D is a fat-soluble secosteroid hormone that exists primarily as cholecalciferol (vitamin D_3_) and ergocalciferol (vitamin D_2_), with D_3_ representing the principal physiological form in humans. Its biological activity depends on sequential hydroxylation steps, including renal conversion of 25-hydroxyvitamin D [25(OH)D] to the active metabolite 1,25-dihydroxyvitamin D [1,25(OH)_2_D], which exerts its effects via the vitamin D receptor (VDR). In clinical practice, vitamin D status is assessed by measuring serum 25(OH)D concentrations [58,59].

In patients with DKD, coexisting vitamin D deficiency, and AH, vitamin D-based interventions should therefore be considered in the context of impaired renal activation and increased cardiometabolic risk. Native vitamin D supplementation remains the most commonly used therapeutic approach. Among available formulations, D_3_ is generally considered preferable to D_2_, as it produces greater and more sustained increases in serum 25(OH)D concentrations. This difference is attributed to the lower affinity of D_2_ for the VDBP and its more rapid clearance, resulting in less stable 25(OH)D exposure [60,61,62].

However, correction of systemic vitamin D deficiency alone may be insufficient in DKD, as impaired renal activation can limit downstream vitamin D signaling. Oral calcifediol allows for more rapid and predictable increases in serum 25(OH)D concentrations and may be advantageous in selected patients, particularly in patients in whom native vitamin D supplementation appears less effective [63]. Active vitamin D metabolites and selective VDR agonists bypass renal 1α-hydroxylation and represent additional options in selected patients. In particular, VDR agonists such as paricalcitol have been shown in randomized trials to be associated with reductions in albuminuria when added to established RAAS blockade; however, evidence regarding long-term renal or cardiovascular outcome modification remains limited. Experimental data also suggest potential beneficial effects on mitochondrial function and oxidative stress in diabetic renal tissue [60,64].

From a hierarchy-of-evidence perspective, mechanistic and experimental findings should be interpreted separately from observational associations and randomized interventional data.

Beyond correction of deficiency, the potential systemic effects of vitamin D-based interventions are also relevant in DKD. Insulin resistance is highly prevalent in this population and contributes to metabolic dysregulation and progression toward type 2 diabetes mellitus. Observational studies have reported that vitamin D supplementation is associated with modest reductions in hyperinsulinemia and improvements in insulin sensitivity [40]. Experimental data suggest that vitamin D may modulate insulin signaling by upregulating insulin receptor expression, enhancing IRS signaling, attenuating inflammatory pathways, and improving skeletal muscle glucose uptake via GLUT4 translocation [58,65]. Nevertheless, meta-analyses of randomized controlled trials have demonstrated only modest and inconsistent improvements in glycemic indices, and large interventional studies have failed to show sustained metabolic benefits, particularly in patients with DKD [58,60]. Thus, while mechanistic and observational data suggest potential metabolic effects, randomized evidence remains inconsistent.

Given the close interplay between metabolic dysfunction and BP regulation in DKD, the potential impact of vitamin D supplementation on AH has also been examined. Several clinical trials in vitamin D-deficient hypertensive patients, including individuals with impaired kidney function, have reported reductions in BP following daily or weekly supplementation with vitamin D or its analogs [5,66]. These findings, however, require cautious interpretation. Many studies are limited by small sample sizes and heterogeneous populations, and impaired kidney function does not necessarily equate to established DKD. Moreover, large randomized controlled trials and meta-analyses have not consistently demonstrated clear or sustained antihypertensive effects of vitamin D supplementation [63]. Overall, the antihypertensive effect appears modest and inconsistent, particularly in patients with established DKD, based on currently available randomized evidence.

Importantly, despite biological plausibility and selected positive findings, current evidence does not support targeted vitamin D supplementation as a disease-modifying intervention for DKD-associated hypertension, particularly in the absence of confirmed vitamin D deficiency.

Despite promising experimental data and selected clinical observations, vitamin D-based interventions in patients with DKD, vitamin D deficiency, and AH are subject to important limitations. Chronic high-dose supplementation may lead to hypercalcemia and hyperphosphatemia, and the optimal upper threshold for serum 25(OH)D concentrations remains uncertain. These concerns are particularly relevant in DKD, where disturbances in mineral metabolism necessitate cautious and individualized supplementation strategies [67].

From a clinical perspective, vitamin D supplementation in DKD should therefore primarily aim at correction of documented deficiency in accordance with CKD guidelines, rather than routine use as an antihypertensive or disease-modifying strategy.

## 4. Discussion

### 4.1. Limitations of the Study

Several limitations should be considered when interpreting this narrative review. Although experimental and observational studies support individual associations between DKD, vitamin D deficiency, and AH, we have not found many studies that examine these three conditions simultaneously within a single, integrated mechanistic framework. As a result, most proposed interactions are based on indirect evidence rather than direct confirmation in unified clinical or experimental models.

The mechanisms discussed involve multiple overlapping renal, metabolic, inflammatory, and vascular processes, making it difficult to clearly separate the effects of vitamin D deficiency from other DKD-related factors.

Evidence regarding vitamin D-based interventions in patients with DKD and AH is heterogeneous, and we have not found consistent data to guide the choice between native vitamin D, active metabolites, or vitamin D receptor agonists.

It is also impossible to determine the patient’s compliance with dietary recommendations and supplementation in most available studies, which limits assessment of exogenous vitamin D intake. In addition, information on the degree of glycemic control in patients with DKD is frequently incomplete or unavailable, making it difficult to account for the influence of diabetes severity and treatment on renal and cardiovascular outcomes.

Finally, current clinical guidelines do not provide specific recommendations for vitamin D supplementation in patients with DKD and AH, reflecting the lack of large, well-designed randomized trials with clinically meaningful renal and cardiovascular outcomes.

### 4.2. Conclusions

Diabetic kidney disease, vitamin D deficiency, and arterial hypertension are common conditions that often coexist and influence one another. In this review, we examined the mechanisms linking these disorders and discussed their implications for disease progression and clinical management.

The available evidence shows that they should not be considered separately, but rather as interrelated processes occurring within the same patients.

Although AH arises from a multifactorial and mosaic pathogenesis, the interactions described in this review highlight the relevance of renal dysfunction and disrupted vitamin D signaling as important contributing elements within this complex network. Given the global burden of DKD, vitamin D deficiency, and AH, understanding these interactions is clinically relevant.

Some questions remain unanswered. There is still a lack of recommendations about vitamin D supplementation specifically tailored to patients with DKD and coexisting AH. In addition, available studies often provide limited information on exogenous vitamin D intake from diet and supplementation, as well as on the level of glycemic control in patients with DKD, which makes interpretation of clinical outcomes difficult. More prospective studies in large groups are necessary to establish the prevalence of these interactions and to determine their clinical significance.

Taken together, viewing DKD, vitamin D deficiency, and AH as elements of a vicious cycle may improve understanding of their coexistence and support a more integrated approach to future research and clinical management. At the same time, the true impact of vitamin D on renal and cardiovascular health remains part of a complex and evolving landscape that requires continued scientific exploration.

## Data Availability

No new data were generated or analyzed in this study.

## Abbreviations

The following abbreviations are used in this manuscript:

DKD	diabetic kidney disease
GFR	glomerular filtration rate
CKD	chronic kidney disease
AH	arterial hypertension
RAAS	renin–angiotensin–aldosterone system
BP	blood pressure
25(OH)D	25-hydroxyvitamin D
1,25(OH)2D	1,25-dihydroxyvitamin D
VDBP	vitamin D- binding protein
FGF-23	fibroblast growth factor 23
Ang I	Angiotensin I
Ang II	Angiotensin II
AT1R	angiotensin II type 1 receptor
CREB	cAMP response element-binding protein
VDR	vitamin D receptor
CREs	cAMP response elements
MR	mineralocorticoid receptor
ROS	reactive oxygen species
IL-1	interleukin-1
IL-6	interleukin-6
CRP	C-reactive protein
MCP-1	monocyte chemoattractant protein-1
TNF-α	tumor necrosis factor alpha
hs-CRP	high-sensitivity C-reactive protein
T2DM	type 2 diabetes mellitus
IR	insulin resistance
GSH	glutathione
NO	nitric oxide
ET-1	endothelin-1
AGEs	advanced glycation end products
IRS	insulin receptor substrate
GLUT4	glucose transporter type

## References

[1] Galuška D., Pácal L., Kaňková K. (2021). Pathophysiological Implication of Vitamin D in Diabetic Kidney Disease. Kidney Blood Press. Res..

[2] Huang H.Y., Lin T.W., Hong Z.X., Lim L.M. (2023). Vitamin D and Diabetic Kidney Disease. Int. J. Mol. Sci..

[3] Zappulo F., Cappuccilli M., Cingolani A., Scrivo A., Chiocchini A.L.C., Nunzio M.D., Donadei C., Napoli M., Tondolo F., Cianciolo G. (2022). Vitamin D and the Kidney: Two Players, One Console. Int. J. Mol. Sci..

[4] National Institutes of Health, Office of Dietary Supplements (2019). Vitamin D—Fact Sheet for Health Professionals.

[5] Secondulfo C., Visco V., Virtuoso N., Fortunato M., Migliarino S., Rispoli A., La Mura L., Stellato A., Caliendo G., Settembre E. (2024). Vitamin D: A Bridge between Kidney and Heart. Life.

[6] Ruilope L.M., Agarwal R., Anker S.D., Filippatos G., Pitt B., Rossing P., Sarafidis P., Schmieder R.E., Joseph A., Rethemeier N. (2022). Blood Pressure and Cardiorenal Outcomes with Finerenone in Chronic Kidney Disease in Type 2 Diabetes. Hypertension.

[7] Leoncini G., Viazzi F., De Cosmo S., Russo G., Fioretto P., Pontremoli R. (2020). Blood Pressure Reduction and RAAS Inhibition in Diabetic Kidney Disease: Therapeutic Potentials and Limitations. J. Nephrol..

[8] Lim K., Hamano T., Thadhani R. (2018). Vitamin D and Calcimimetics in Cardiovascular Disease. Semin. Nephrol..

[9] Zand L., Kumar R. (2017). The Use of Vitamin D Metabolites and Analogues in the Treatment of Chronic Kidney Disease. Endocrinol. Metab. Clin. N. Am..

[10] Afzal S., Nordestgaard B.G. (2017). Vitamin D, Hypertension, and Ischemic Stroke in 116 655 Individuals from the General Population: A Genetic Study. Hypertension.

[11] Gluba-Brzózka A., Franczyk B., Ciałkowska-Rysz A., Olszewski R., Rysz J. (2018). Impact of Vitamin D on the Cardiovascular System in Advanced Chronic Kidney Disease (CKD) and Dialysis Patients. Nutrients.

[12] Gois P.H.F., Wolley M., Ranganathan D., Seguro A.C. (2018). Vitamin D Deficiency in Chronic Kidney Disease: Recent Evidence and Controversies. Int. J. Environ. Res. Public Health.

[13] Latic N., Erben R.G. (2020). Vitamin D and Cardiovascular Disease, with Emphasis on Hypertension, Atherosclerosis, and Heart Failure. Int. J. Mol. Sci..

[14] Brosolo G., Da Porto A., Bulfone L., Scandolin L., Vacca A., Bertin N., Vivarelli C., Sechi L.A., Catena C. (2022). Vitamin D Deficiency Is Associated with Glycometabolic Changes in Nondiabetic Patients with Arterial Hypertension. Nutrients.

[15] Jean G., Souberbielle J.C., Chazot C. (2017). Vitamin D in Chronic Kidney Disease and Dialysis Patients. Nutrients.

[16] Charchar F.J., Prestes P.R., Mills C., Ching S.M., Neupane D., Marques F.Z., Sharman J.E., Vogt L., Burrell L.M., Korostovtseva L. (2024). Lifestyle Management of Hypertension: International Society of Hypertension Position Paper Endorsed by the World Hypertension League and European Society of Hypertension. J. Hypertens..

[17] Colbert G.B., Elrggal M.E., Gaddy A., Madariaga H.M., Lerma E.V. (2023). Management of Hypertension in Diabetic Kidney Disease. J. Clin. Med..

[18] Jhee J.H., Nam K.H., An S.Y., Cha M.U., Lee M., Park S., Kim H., Yun H.R., Kee Y.K., Park J.T. (2018). Severe Vitamin D Deficiency Is a Risk Factor for Renal Hyperfiltration. Am. J. Clin. Nutr..

[19] Jørgensen H.S., Vervloet M., Cavalier E., Bacchetta J., de Borst M.H., Bover J., Cozzolino M., Ferreira A.C., Hansen D., Herrmann M. (2025). The Role of Nutritional Vitamin D in Chronic Kidney Disease–Mineral and Bone Disorder in Children and Adults with Chronic Kidney Disease, on Dialysis, and after Kidney Transplantation—A European Consensus Statement. Nephrol. Dial. Transplant..

[20] Uwaezuoke S.N. (2021). Vitamin D Analogs Can Retard the Onset or Progression of Diabetic Kidney Disease: A Systematic Review. Front. Clin. Diabetes Healthc..

[21] Legarth C., Grimm D., Wehland M., Bauer J., Krüger M. (2018). The Impact of Vitamin D in the Treatment of Essential Hypertension. Int. J. Mol. Sci..

[22] Govender D., Damjanovic L., Gaza C.A., Meyer V. (2021). Vitamin D Decreases Silencer Methylation to Downregulate Renin Gene Expression. Gene.

[23] Jensen N.S., Wehland M., Wise P.M., Grimm D. (2023). Latest Knowledge on the Role of Vitamin D in Hypertension. Int. J. Mol. Sci..

[24] Gembillo G., Siligato R., Amatruda M., Conti G., Santoro D. (2021). Vitamin D and Glomerulonephritis. Medicina.

[25] Taheri S., Asim M., al Malki H., Fituri O., Suthanthiran M., August P., Rachid A., Adel A., Hamdi A., Nauman A. (2018). Intervention Using Vitamin D for Elevated Urinary Albumin in Type 2 Diabetes Mellitus (IDEAL-2 Study): Study Protocol for a Randomised Controlled Trial. Trials.

[26] Sugahara M., Pak W.L.W., Tanaka T., Tang S.C.W., Nangaku M. (2021). Update on Diagnosis, Pathophysiology, and Management of Diabetic Kidney Disease. Nephrology.

[27] Renke G., Starling-Soares B., Baesso T., Petronio R., Aguiar D., Paes R. (2023). Effects of Vitamin D on Cardiovascular Risk and Oxidative Stress. Nutrients.

[28] Humle K., Klanger B., Kolkhof P., Rosas S.E., Rossing P., Wright E., Jefferson N. (2024). Summary of Research: Cardiovascular and Kidney Outcomes with Finerenone in Patients with Type 2 Diabetes and Chronic Kidney Disease—The FIDELITY Pooled Analysis. Diabetes Ther..

[29] de Bhailis Á.M., Kalra P.A. (2022). Hypertension and the Kidneys. Br. J. Hosp. Med..

[30] Samsu N. (2021). Diabetic Nephropathy: Challenges in Pathogenesis, Diagnosis, and Treatment. BioMed Res. Int..

[31] Ricciardi C.A., Gnudi L. (2021). Kidney Disease in Diabetes: From Mechanisms to Clinical Presentation and Treatment Strategies. Metabolism.

[32] Unger T., Borghi C., Charchar F., Khan N.A., Poulter N.R., Prabhakaran D., Ramirez A., Schlaich M., Stergiou G.S., Tomaszewski M. (2020). 2020 International Society of Hypertension Global Hypertension Practice Guidelines. Hypertension.

[33] Kim H.L. (2023). Arterial Stiffness and Hypertension. Clin. Hypertens..

[34] Arendshorst W.J., Vendrov A.E., Kumar N., Ganesh S.K., Madamanchi N.R. (2024). Oxidative Stress in Kidney Injury and Hypertension. Antioxidants.

[35] Lin L., Tan W., Pan X., Tian E., Wu Z., Yang J. (2022). Metabolic Syndrome-Related Kidney Injury: A Review and Update. Front. Endocrinol..

[36] Dwivedi S., Sikarwar M.S. (2024). Diabetic Nephropathy: Pathogenesis, Mechanisms, and Therapeutic Strategies. Horm. Metab. Res..

[37] Zoccali C., Mallamaci F., Adamczak M., De Oliveira R.B., Massy Z.A., Sarafidis P., Agarwal R., Mark P.B., Kotanko P., Ferro C.J. (2023). Cardiovascular Complications in Chronic Kidney Disease: A Review from the European Renal and Cardiovascular Medicine Working Group of the European Renal Association. Cardiovasc. Res..

[38] Drożdż D., Drożdż M., Wójcik M. (2023). Endothelial Dysfunction as a Factor Leading to Arterial Hypertension. Pediatr. Nephrol..

[39] Guo W., Song Y., Sun Y., Du H., Cai Y., You Q., Fu H., Shao L. (2022). Systemic Immune-Inflammation Index Is Associated with Diabetic Kidney Disease in Type 2 Diabetes Mellitus Patients: Evidence from NHANES 2011-2018. Front. Endocrinol..

[40] Argano C., Mirarchi L., Amodeo S., Orlando V., Torres A., Corrao S. (2023). The Role of Vitamin D and Its Molecular Bases in Insulin Resistance, Diabetes, Metabolic Syndrome, and Cardiovascular Disease: State of the Art. Int. J. Mol. Sci..

[41] Contreras-bol V., Garc B., Garc C., Muñoz-torres M. (2021). Mechanisms Involved in the Relationship between Vitamin D and Insulin Resistance: Impact on Clinical Practice. Nutrients.

[42] Angeli F., Reboldi G., Verdecchia P. (2021). The Link between Inflammation and Hypertension: Unmasking Mediators. Am. J. Hypertens..

[43] Griendling K.K., Camargo L.L., Rios F.J., Alves-Lopes R., Montezano A.C., Touyz R.M. (2021). Oxidative Stress and Hypertension. Circ. Res..

[44] Kaur G., Singh J., Kumar J. (2019). Vitamin D and Cardiovascular Disease in Chronic Kidney Disease. Pediatr. Nephrol..

[45] Wang S., Xu L., Wu Y., Shen H., Lin Z., Fang Y., Zhang L., Shen B., Liu Y., Wu K. (2022). Parathyroid Hormone Promotes Human Umbilical Vein Endothelial Cell Migration and Proliferation Through Orai1-Mediated Calcium Signaling. Front. Cardiovasc. Med..

[46] Islam H., Hassaan S.M., Islam R., Islam T., Zaidi F., Rehman H.U., Haque M.M.U., Turabee Z., Asim M., Ahmad I. (2024). Vitamin D’s Role in Cardiovascular Diseases. Discov. Med..

[47] Durante A., Mazzapicchi A., Baiardo Redaelli M. (2024). Systemic and Cardiac Microvascular Dysfunction in Hypertension. Int. J. Mol. Sci..

[48] Vadana M., Cecoltan S., Ciortan L., Macarie R.D., Mihaila A.C., Tucureanu M.M., Gan A.M., Simionescu M., Manduteanu I., Droc I. (2022). Parathyroid Hormone Induces Human Valvular Endothelial Cells Dysfunction That Impacts the Osteogenic Phenotype of Valvular Interstitial Cells. Int. J. Mol. Sci..

[49] Szymczak-Pajor I., Śliwińska A. (2019). Analysis of Association between Vitamin D Deficiency and Insulin Resistance. Nutrients.

[50] Kosmas C.E., Bousvarou M.D., Kostara C.E., Papakonstantinou E.J., Salamou E., Guzman E. (2023). Insulin Resistance and Cardiovascular Disease. J. Int. Med. Res..

[51] Szymczak-Pajor I., Drzewoski J., Śliwińska A. (2020). The Molecular Mechanisms by Which Vitamin D Prevents Insulin Resistance and Associated Disorders. Int. J. Mol. Sci..

[52] Lei X., Zhou Q., Wang Y., Fu S., Li Z., Chen Q. (2023). Serum and Supplemental Vitamin D Levels and Insulin Resistance in T2DM Populations: A Meta-Analysis and Systematic Review. Sci. Rep..

[53] Bao K., Bao D., Huang Z., Gu W., Chen K. (2025). Associations of Insulin Resistance Surrogate with Resistant Hypertension and the Severity of Hypertension in the Chronic Kidney Disease Population. J. Hypertens..

[54] Wang N., Li J., Tian E., Li S., Liu S., Cao F., Kong J., Yue B. (2025). Renin-Angiotensin-Aldosterone System Variations in Type 2 Diabetes Mellitus Patients with Different Complications and Treatments: Implications for Glucose Metabolism. PLoS ONE.

[55] Wahba N.S., Abdel-Ghany R.H., Ghareib S.A., Abdel-Aal M., Alsemeh A.E. (2020). Vitamin D3 Potentiates the Renoprotective Effects of Vildagliptin in a Rat Model of Fructose/Salt-Induced Insulin Resistance. Eur. J. Pharm. Sci..

[56] Theodorakis N., Nikolaou M. (2025). From Cardiovascular-Kidney-Metabolic Syndrome to Cardiovascular-Renal-Hepatic-Metabolic Syndrome: Proposing an Expanded Framework. Biomolecules.

[57] Valente V., Izzo R., Manzi M.V., de Luca M.R., Barbato E., Morisco C. (2021). Modulation of Insulin Resistance by Renin Angiotensin System Inhibitors: Implications for Cardiovascular Prevention. Monaldi Arch. Chest Dis..

[58] Wu J., Atkins A., Downes M., Wei Z. (2023). Vitamin D in Diabetes: Uncovering the Sunshine Hormone’s Role in Glucose Metabolism and Beyond. Nutrients.

[59] Mourelatou N.G., Kounatidis D., Jude E.B., Rebelos E. (2024). Vitamin D Supplementation as a Therapeutic Strategy in Autoimmune Diabetes: Insights and Implications for LADA Management. Nutrients.

[60] Sharma J.K., Khan S., Wilson T., Pilkey N., Kapuria S., Roy A., Adams M.A., Holden R.M. (2023). Are There Any Pleiotropic Benefits of Vitamin D in Patients With Diabetic Kidney Disease? A Systematic Review of Randomized Controlled Trials. Can. J. Kidney Health Dis..

[61] van den Heuvel E.G., Lips P., Schoonmade L.J., Lanham-New S.A., van Schoor N.M. (2024). Comparison of the Effect of Daily Vitamin D2 and Vitamin D3 Supplementation on Serum 25-Hydroxyvitamin D Concentration (Total 25(OH)D, 25(OH)D2, and 25(OH)D3) and Importance of Body Mass Index: A Systematic Review and Meta-Analysis. Adv. Nutr..

[62] Zhou S., Glowacki J. (2017). Chronic Kidney Disease and Vitamin D Metabolism in Human Bone Marrow–Derived MSCs. Ann. N. Y. Acad. Sci..

[63] de la Guía-Galipienso F., Martínez-Ferran M., Vallecillo N., Lavie C.J., Sanchis-Gomar F., Pareja-Galeano H. (2021). Vitamin D and Cardiovascular Health. Clin. Nutr..

[64] Chen H., Zhang H., Li A.-M., Liu Y.-T., Liu Y., Zhang W., Yang C., Song N., Zhan M., Yang S. (2024). VDR Regulates Mitochondrial Function as a Protective Mechanism against Renal Tubular Cell Injury in Diabetic Rats. Redox Biol..

[65] Chen W., Liu L., Hu F. (2024). Efficacy of Vitamin D Supplementation on Glycaemic Control in Type 2 Diabetes: An Updated Systematic Review and Meta-Analysis of Randomized Controlled Trials. Diabetes Obes. Metab..

[66] Chen S., Gemelga G., Yeghiazarians Y. (2022). Is Vitamin D Supplementation an Effective Treatment for Hypertension?. Curr. Hypertens. Rep..

[67] Zhou F., Jamilian A., Prabahar K., Hernández-Wolters B., Kord-Varkaneh H., Bai D. (2024). The Effect of Vitamin D2 Supplementation on Vitamin D Levels in Humans: A Time and Dose–Response Meta-Analysis of Randomized Controlled Trials. Steroids.

